# Empagliflozin, a sodium glucose co-transporter-2 inhibitor, alleviates atrial remodeling and improves mitochondrial function in high-fat diet/streptozotocin-induced diabetic rats

**DOI:** 10.1186/s12933-019-0964-4

**Published:** 2019-11-28

**Authors:** Qingmiao Shao, Lei Meng, Sharen Lee, Gary Tse, Mengqi Gong, Zhiwei Zhang, Jichao Zhao, Yungang Zhao, Guangping Li, Tong Liu

**Affiliations:** 1 Tianjin Key Laboratory of Ionic-Molecular Function of Cardiovascular Disease, Department of Cardiology, Tianjin Institute of Cardiology, Second Hospital of Tianjin Medical University, Tianjin, 300211 People’s Republic of China; 2 Laboratory of Cardiovascular Physiology, Li Ka Shing Institute of Health Sciences, Sha Tin, Hong Kong S.A.R., China; 3 Auckland Bioengineering Institute, The University of Auckland, Auckland, New Zealand; 4 Tianjin Key Laboratory of Exercise Physiology and Sports Medicine, Department of Health & Exercise Science, Tianjin University of Sport, Tianjin, 300381 People’s Republic of China

**Keywords:** Atrial fibrillation, Empagliflozin, SGLT-2 inhibitor, Atrial remodeling, Mitochondrial function, Diabetic rats

## Abstract

**Background:**

Diabetes mellitus is an important risk factor for atrial fibrillation (AF) development. Sodium–glucose co-transporter-2 (SGLT-2) inhibitors are used for the treatment of type 2 diabetes mellitus (T2DM). Their cardioprotective effects have been reported but whether they prevent AF in T2DM patients are less well-explored. We tested the hypothesis that the SGLT-2 inhibitor, empagliflozin, can prevent atrial remodeling in a diabetic rat model.

**Methods:**

High-fat diet and low-dose streptozotocin (STZ) treatment were used to induce T2DM. A total of 96 rats were randomized into the following four groups: (i) control (ii) T2DM, (iii) low-dose empagliflozin (10 mg/kg/day)/T2DM; and (iv) high-dose empagliflozin (30 mg/kg/day)/T2DM by the intragastric route for 8 weeks.

**Results:**

Compared with the control group, left atrial diameter, interstitial fibrosis and the incidence of AF inducibility were significantly increased in the DM group. Moreover, atrial mitochondrial respiratory function, mitochondrial membrane potential, and mitochondrial biogenesis were impaired. Empagliflozin treatment significantly prevented the development of these abnormalities in DM rats, likely via the peroxisome proliferator-activated receptor-c coactivator 1α (PGC-1α)/nuclear respiratory factor-1 (NRF-1)/mitochondrial transcription factor A (Tfam) signaling pathway.

**Conclusions:**

Empagliflozin can ameliorate atrial structural and electrical remodeling as well as improve mitochondrial function and mitochondrial biogenesis in T2DM, hence may be potentially used in the prevention of T2DM-related atrial fibrillation.

## Introduction

Atrial fibrillation (AF) is the commonest type of sustained cardiac arrhythmia with an age-related increase in its prevalence, affecting around 5.5% of the European population above the age of 55 [1]. Diabetes mellitus (DM) predisposes to AF development by inducing structural and electrical remodelling in the atria [2, 3]. Moreover, AF is associated with a substantial increase in the risk of cardiovascular events and death in DM patients [2, 3].

Recently there have been numerous trial and real-world studies on the cardiovascular benefits of sodium–glucose co-transporter-2 (SGLT-2) inhibitors in type 2 diabetic patients. Of these, the EMPAgliflozin Removal of Excess of Glucose OUTCOME (EMPA-REG OUTCOME) trial was a randomized controlled trial examining the empagliflozin in patients with both type 2 diabetes mellitus (T2DM) and established cardiovascular disease (CVD). In this study, empagliflozin reduced the composite endpoint of cardiovascular death, nonfatal myocardial infarction or nonfatal stroke, in addition to death and heart failure-related hospitalizations [4]. Similar reductions in cardiovascular deaths and HF burden were also found in the CANagliflozin cardioVascular Assessment Study (CANVAS) program, which evaluated the effectiveness of canagliflozin in 10,142 patients with type 2 DM [5, 6]. However, the impact of SGLT-2 inhibition in atrial remodeling remains unclear [7]. In this study, we investigated the effects of empagliflozin (EMPA), a commercially available and highly selective SGLT-2 inhibitor, on atrial remodeling in high-fat diet (HFD)/streptozotocin (STZ)-induced diabetic rats.

## Methods

### Experimental animals and protocol

This study conformed to the National Research Council (US) Committee for the Update of the Guide for the Care and Use of Laboratory Animals. It was approved by the Experimental Animal Administration Committee of Tianjin Medical University and Tianjin Municipal Commission for Experimental Animal Control.

HFD and low-dose STZ treatment were used to induce type II diabetes mellitus in rats [8]. The HFD + STZ model, which similar demonstrates a progression from insulin resistance to hypoinsulinaemia and hyperglycemia, mimics the natural T2DM pathogenesis in humans and is suitable to investigate the pathogenesis of diabetic complications and test the efficiency of anti-diabetic agents. A total of 96 adult male Sprague–Dawley (SD) rats (200 ± 20 g) were purchased from the HuaFuKang Bioscience Co., LTD (Beijing, China). They were kept under a 12 h light/dark cycle at room temperature (20–22 °C) and humidity (50–60%). After 1 week, the rats were divided into two groups: HFD group (n = 72) and control group (n = 24). The rats of the HFD group were fed high-fat chow (H10060, fat energy ratio = 60 kcal%, protein energy ratio = 20 kcal%, carbohydrate energy ratio = 20 kcal%, the Beijing HuaFuKang Bioscience Co., LTD, China) for 4 weeks, then given a single tail vein injection of STZ (30 mg/kg; Sigma-Aldrich, St. Louis, MO, USA) dissolved in citrate buffer at pH 4.5. The rats of control group were fed regular chow and were injected with the same dose of citrate buffer. One week following the STZ injection, blood samples were collected from the tail vein to measure the blood glucose level. The blood glucose level of control group rats was kept within the normal range. HFD group rat with random blood glucose > 16.7 mmol/L was considered successful induction of DM, and was used for further investigation [9]. The same dose of STZ was injected again in rats with blood glucose level that did not meet the diagnostic criteria. The remaining rats that failed to meet the diagnostic criteria after the injection were excluded from the study. This process was repeated until a sufficient number of DM animals were produced. Blood glucose concentration of the DM models was monitored weekly using the glucometer Optium Xceed (Abbott Laboratories MediSense Products).

The rats were then divided into four groups: control group (CON, n = 24); DM group (DM, 0.5% hydroxyethylcellulose/day, intragastric administration, ig, n = 24); low dose of EMPA (low-EMPA, 10 mg/kg/day, ig, n = 24); and high dose of EMPA (high-EMPA, 30 mg/kg/day, ig, n = 24). The dose of empagliflozin was based on the previous studies [10, 11]. Empagliflozin was supplied by Boehringer Ingelheim Pharma GmbH & Co. (KG, Germany). Besides from the control group, the remaining three groups were composed of the DM models. The rats were treated for 8 weeks. All rats were anesthetized with sodium pentobarbital by intraperitoneal injection and sacrificed following weeks of treatment. The first 8 rats of each group were used for the first part of the experiments (including echocardiographic, hemodynamic, histological, and serum biochemical and oxidative stress-related markers examination, and western blot analysis). The next eight rats were used for the electrophysiological studies, and the remaining eight rats were used for examinations of mitochondrial function.

### Echocardiographic assessment

After 8 weeks, transthoracic echocardiography was performed by blinded operators. The rats were anesthetized, which was induced by inhaling 3% isoflurane (ISO) and maintained at a lower dose (1.5–2% ISO) subsequently. Then the rats were placed on the table in the horizontal position. Echocardiographic parameters were obtained in the parasternal long-axis view using a small animal ultrasound system (Visual Sonics Vevo 2100, SONICS, Newtown, CT, USA). Left atrial (LA) anteroposterior diameter, left ventricular posterior wall thickness (LVPWT), interventricular septal thickness (IVST), left ventricular end-diastolic dimension (LVEDD), and left ventricular end systolic dimension (LVESD) were measured using 2-dimensional imaging during 5 consecutive cardiac cycles. Left ventricular ejection fraction (LVEF) was calculated using the following formulae:

$$ {\text{LVEDV}}\left( {{\text{left}}\;{\text{ventricular}}\;{\text{end}} - {\text{diastolic}}\;{\text{volume}}} \right)\, = \,\left[ {7/\left( {2.4\, + \,{\text{LVEDD}}} \right)} \right]\, \times \,{\text{LVEDD}}^{3} ; $$

$$ {\text{LVESV}}\left( {{\text{left}}\;{\text{ventricular}}\;{\text{end}} - {\text{systolic}}\;{\text{volume}}} \right)\, = \,\left[ {7/\left( {2.4\, + \,{\text{LVESD}}} \right)} \right]\, \times \,{\text{LVESD}}^{3} ; $$

$$ {\text{LVEF}}\, = \,\left[ {\left( {{\text{LVEDV}} - {\text{LVESV}}} \right)/{\text{LVEDV}}} \right]\, \times \,100\% . $$

Three repeated measurements of each parameter were averaged for subsequent analysis.

### Hemodynamic studies

At the end of echocardiographic examination, each rat underwent right carotid artery cannulation allowing measurements of hemodynamic parameters using Millar catheter during electrocardiographic (ECG) monitoring. Heart rate, aortic systolic blood pressure (SBP), diastolic blood pressure (DBP), and mean blood pressure (MBP) were recorded carefully after a stabilization period. A cannula was then inserted through the aortic valve to the left ventricle to measure the ventricular end-diastolic pressure, in addition to the maximal and minimal rates of the rise in left ventricular pressure.

### Sample collection and storage

While completing the hemodynamic examination, the rats were anesthetized with sodium pentobarbital. Hearts were quickly removed and rinsed with phosphate-buffered saline. Left atrial tissues used for histological study were fixed in 10% formaldehyde for 3 days, and then embedded in paraffin after dehydration. Other left atrial tissues were rapidly frozen in liquid nitrogen for protein analysis.

### Serum biochemical, inflammatory and oxidative stress markers measurements

Fasting glucose, total cholesterol (TC), triglycerides (TG), low-density lipoprotein cholesterol (LDL-C), high-density lipoprotein cholesterol (HDL-C), and creatinine (Cr) level were detected using a full automatic biochemical analyzer at the 8th week. Insulin (INS), brain natriuretic peptide (BNP) and high-sensitivity C-reactive protein (hs-CRP) were assessed using the rat ELISA Kits (Wuhan Huamei Biological Engineering Co, Ltd, China). A lipid peroxidation malondialdehyde (MDA) and superoxide dismutase (SOD) assay kit (Nanjing Jianchen Bioengineering Institute, China) were used to detect the serum MDA and SOD level.

### Histological analyses

The LA myocardium was cut at 4 μm intervals, then stained with hematoxylin and eosin (H&E), and Masson’s trichrome stains to evaluate the cardiomyocyte diameter, and the extent of interstitial fibrosis respectively. All slices were observed and photographed under 40× objective. The cell borders were measured in the short-axis view with a visible mononucleus from 10 random fields. An average of 40 cardiomyocytes per animal was analyzed from each animal in the group. Micrographs were digitized using Adobe Photoshop (Version 7.0). To quantify the areas of interstitial fibrosis in the LA myocardium, the blue pixel content of the digitized images, excluding the perivascular fibrotic areas, was measured relative to the total tissue area using Image-Pro Plus 6.0 Scion image software (Scion Corporation).

### Isolated heart electrophysiological studies

To evaluate the electrophysiological properties of rat hearts, median sternotomy was performed under anesthetization with 3% sodium pentobarbital (30 mg/kg), and the hearts were quickly removed. The Langendorff heart perfusion technique was used for retrograde perfusion of the heart via the aorta. Three pairs of electrodes were inserted into the high right atrium, high left atrium, and right ventricle. Heart rate (HR), interatrial conduction time (IACT), high right atrial effective refractory period (HRAERP), high left atrial effective refractory period (HLAERP), and AF inducibility were measured. AF induction was tested by burst pacing (cycle length of 50 ms, 40 ms, 30 ms, and 25 ms) for 3 s respectively, which was performed 5 times with 30-s intervals. AF was defined as rapid, irregular atrial response longer than 1 s.

### Isolation of mitochondria from atrial tissue

Rats were euthanized with 3% sodium pentobarbital and median sternotomy was immediately performed. A portion of 100 mg of the left atrial tissue was quickly dissected and minced in an ice-cold isolation medium (sucrose 17.115 g, HEPES 0.143 g, EDTA Na_2_ 0.037 g, distilled water 200 mL and PH 7.4). The minced blood-free tissue was homogenized using a manual glass homogenizer with 6 passes (0–4 °C). Subsequently, the homogenate was centrifuged at 1000*g* for 10 min and the liquid supernatant was collected, which was then centrifuged at 10,000*g* for 10 min. The major constituent of the deposit was mitochondrial pellet, which was suspended in 0.5 mL of the conversational medium (KCL 1.928 g, HEPES 0.143 g, EDTA Na_2_ 0.037 g, KH_2_PO4 0.054 g, BSA 0.2 g, distilled water 200 mL and PH 7.4). The mitochondrial isolation procedures were completed within 1 h after the rats were euthanized. Mitochondrial protein content was assayed using a BSA protein assay reagent kit (Thermo Scientific).

### Quantification of mitochondrial respiration function

Mitochondrial respiratory function was measured polarographically at 25 °C using a Clark-type oxygen electrode (Oroboros Instruments). After an equilibration period, 300 μg of mitochondrial protein was added to the reaction system. Upon stabilization of the mitochondrial oxygen consumption, a 20 μL mixture of 0.8 mol/L malic acid and 1 mol/L glutamic acid was added to initiate the state 2 respiration. After stable state 2 respiration was established, state 3 respiration was initiated by the addition of 20 μL 0.5 mol/L adenosine diphosphate (ADP). When all of the ADP had been phosphorylated to adenosine triphosphate (ATP), the respiratory rate returned to state 4. The respiratory control ratio was calculated as the ratio of the respiratory rate in state 3 to that in state 4.

### Mitochondrial membrane potential measurements

Mitochondrial membrane potential (Dw) was assessed with tetraethyl benzimidazolyl carbocyanine iodide cationic dye, which exhibited potential-dependent accumulation in mitochondria, resulting in a fluorescence emission shift from 525 nm (green) to 590 nm (red) (Cary Eclipse fluorescence spectrophotometer, Varian companies in the United States). Therefore, loss of Dw was detectable by the decrease in the red to green fluorescence emission ratio [12]. The experiments were conducted at 25 °C in 2 mL of respiration medium with 300 μg of mitochondrial protein, and tetraethyl benzimidazolyl carbocyanine iodide dye equilibration was allowed for 10 min. Mitochondrial respiratory function was initiated by a 15 μL mixture of 0.8 mol/L malic acid and 1 mol/L glutamic acid, and the alteration of the fluorescence emission was detected.

### Western blot analysis

Total protein was extracted with RIPA lysate (KeyGEN BioTECH, Nanjing, China) and phenylmethylsulfonyl fluoride (PMSF; KeyGEN BioTECH). Samples (60 μg, 5–10 μL) were run on an SDS-PAGE gel followed by blotting to a polyvinylidene fluoride (PVDF) membrane. After blocking with 5% skim milk, the membranes were incubated with the following primary antibodies: β-actin (1:5000 Abcam, Branford, CT, USA), PGC-1a (1:1000; Abcam), NRF-1 (1:1000; Abcam), and Tfam (1:2000; Abcam), fusion protein mitofusin 1 (Mfn-1) (1:2000; Abcam), optic atrophy 1 (OPA-1) (1:1000; Abcam) and dynamin-related protein 1 (DRP-1) (1:500; Abcam), followed by incubation with appropriate secondary antibodies. Rhea ECL (US Everbright Inc., Suzhou, China) was used as the developer reagent, and the band intensity was assessed by using Image lab software and referenced to β-actin.

### Statistical analysis

Data were presented as mean ± standard deviation (SD). Comparisons among the 4 groups were analyzed for statistical significance using one-way ANOVA followed by Bonferroni correction for comparisons between 2 groups. SPSS 22.0 statistical software was employed for data analysis, and a p value < 0.05 was considered statistically significant.

## Results

### Echocardiographic and hemodynamic studies

**Fig. 1:**
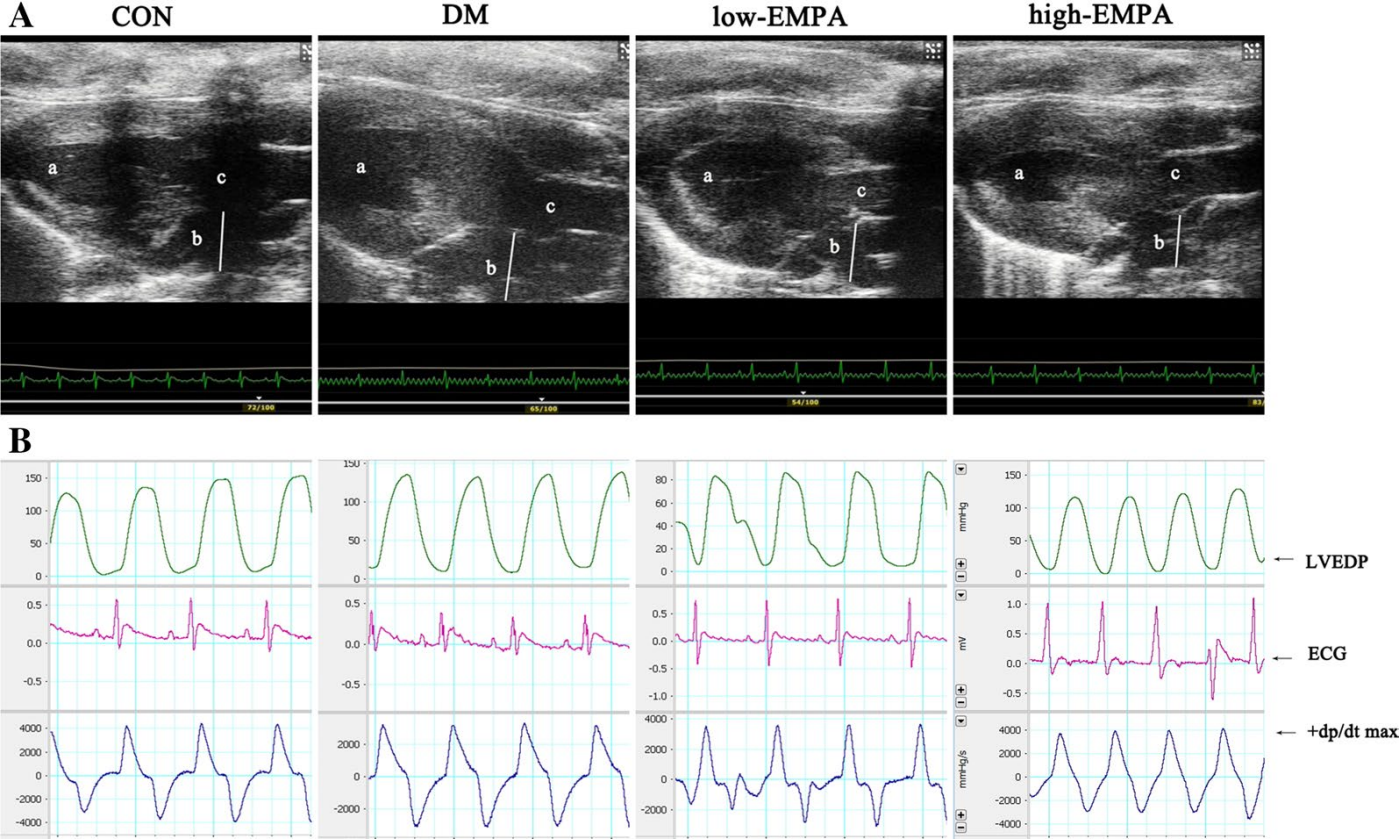
Representative echocardiographic imaging of the atria obtained during left ventricular end-systole and hemodynamic images from the four different groups. **A** Representative measurements echocardiographic imaging of the left atria in control group, diabetes mellitus group, low-dose empagliflozin group and high-dose empagliflozin group. a, left ventricle; b, left atria; c, ascending aorta. **B** Representative hemodynamic imaging for the four study groups. Each group included 8 rats

Representative echocardiographic images of the atria and hemodynamic images from the four groups are shown in Fig. 1. Compared with the control group, the LAD, IVST, and LVPWT were significantly increased in the DM group (p < 0.05). In the high-dose empagliflozin group, LAD, IVST, and LVPWT were significantly decreased compared with the DM group (p < 0.05), whilst no significant difference was observed after low-dose empagliflozin treatment (p > 0.05). Moreover, no difference in LVEDD, LVESD, LVEF and FS was observed at either EMPA dose. Hemodynamic studies revealed that the SBP, DBP, MBP, and LVEDP were significantly increased in the T2DM compared to the control rats (p < 0.05), but empagliflozin at either dose did not significantly improve these parameters (p > 0.05). Table 1 summarizes the baseline characteristics of echocardiographic and hemodynamic studies.

**Table 1:** Echocardiographic and hemodynamic studies

	Control group (n = 8)	DM group (n = 8)	Low-EMPA group (n = 8)	High-EMPA group (n = 8)	p value
LAD, mm	4.12 ± 0.24	4.60 ± 0.44*	4.44 ± 0.08*	4.22 ± 0.14**	0.006
IVST, mm	1.62 ± 0.17	1.98 ± 0.14*	1.84 ± 0.06	1.67 ± 0.44**	0.021
LVPWT, mm	1.60 ± 0.17	2.00 ± 0.17*	1.80 ± 0.31	1.67 ± 0.38**	0.028
LVEDD, mm	7.39 ± 0.29	7.16 ± 0.38	7.47 ± 0.32	7.11 ± 0.22	0.530
LVESD, mm	3.73 ± 0.36	4.07 ± 0.49	3.84 ± 0.49	3.99 ± 0.25	0.680
LVEF, %	79.27 ± 3.87	72.49 ± 6.93	77.78 ± 8.66	73.59 ± 3.67	0.381
FS, %	49.50 ± 4.08	43.09 ± 5.87	48.33 ± 8.75	43.85 ± 3.28	0.372
HR, beats per min	308.75 ± 7.66	311.87 ± 4.79	312.00 ± 6.02	310.86 ± 2.10	0.626
SBP, mmHg	125.30 ± 12.95	139.05 ± 14.41*	138.80 ± 6.48*	137.80 ± 4.19*	0.037
DBP, mmHg	75.71 ± 12.19	102.09 ± 7.99*	97.64 ± 6.46*	96.11 ± 5.06*	<0.001
MBP, mmHg	91.06 ± 9.65	114.45 ± 10.01*	111.28 ± 6.11*	110.84 ± 5.34*	<0.001
LVEDP, mmHg	7.78 ± 1.19	9.45 ± 1.33*	8.66 ± 0.81	8.53 ± 0.68	0.028
+dp/dtmax, mmHg/m	4448.00 ± 835.01	3698.00 ± 380.14*	3695.25 ± 384.95*	4198.00 ± 673.63	0.041
−dp/dtmax, mmHg/m	− 2413.32 ± 276.74	− 1538.32 ± 278.87*	− 1592.07 ± 271.84*	− 2004.70 ± 162.30**	< 0.001
BW, kg	0.58 ± 0.05	0.67 ± 0.07*	0.62 ± 0.08	0.54 ± 0.06**	0.018
Heart weight ratio (1/1000)	3.24 ± 0.42	3.59 ± 0.75*	3.35 ± 0.54	3.12 ± 0.65**	0.451

Values are expressed as mean ± SD. Heart weight ratio indicates the ratio of heart weight and body weight

*DM* diabetes mellitus, *LAD* left atrial diameter, *IVST* interventricular septum, *LVPWT* left ventricular posterior wall, *LVEDD* left ventricular end-diastolic dimension, *LVESD* left ventricular end-systolic dimension, *LVEF* left ventricular ejection fraction, *FS* fraction shortening, *HR* heart rate, *SBP* systolic blood pressure, *DBP* diastolic blood pressure, *MBP* mean blood pressure, *LVEDP* left ventricular end diastolic pressure, *+dp/dtmax* maximal increasing rate of left intraventricular pressure, *−dp/dtmax* maximal decreasing rate of left intraventricular pressure, *BW* body weight

* Compared with the control group, p < 0.05

** Compared with the DM group, p < 0.05

### Serum biochemical, inflammatory and oxidative stress-related examination

Fasting glucose, TG and TC levels at the 8th week were the highest in the untreated DM group compared to the control group (Table 2). Empagliflozin treatment significantly lower fasting glucose, TG and TC level in T2DM rats (p < 0.01). Whilst there was a tendency of higher serum insulin concentration in the DM group compared to controls, this difference was not statistically significant (p > 0.05). The levels of Cr, LDL-C, HDL-C and BNP were not significantly different amongst the four study groups. Increased hs-CRP and MDA concentrations and decreased SOD activity were observed in the DM group compared to the control group (p < 0.05). Both low- and high-dose empagliflozin significantly restored SOD activity (p < 0.05) whilst only high-dose empagliflozin restored hs-CRP and MDA (p < 0.05).

**Table 2:** Serum biochemical, oxidative stress, and inflammation examination

	Control group (n = 8)	DM group (n = 8)	low-EMPA group (n = 8)	high-EMPA group (n = 8)	p value
Glucose, mmol/L	6.38 ± 0.99	21.10 ± 6.71*	13.81 ± 2.19**	12.93 ± 3.39**	< 0.001
Insulin, mIU/L	44.66 ± 8.66	84.66 ± 17.38	70.83 ± 16.93	69.50 ± 11.49	0.135
Creatinine, μmol/L	56.50 ± 8.00	55.14 ± 11.51	51.10 ± 11.84	56.83 ± 10.96	0.761
BUN, mmol/L	7.99 ± 1.34	7.29 ± 1.58	6.57 ± 0.47	7.34 ± 2.05	0.348
TC, mmol/L	1.36 ± 0.42	2.21 ± 0.33*	1.73 ± 0.45**	1.47 ± 0.31**	0.003
Triglycerides, mmol/L	0.57 ± 0.27	0.95 ± 0.32*	0.72 ± 0.58**	0.70 ± 0.40**	0.041
LDL-C, mmol/L	0.44 ± 0.14	0.61 ± 0.13	0.40 ± 0.38	0.48 ± 0.2	0.212
HDL-C, mmol/L	0.96 ± 0.28	0.98 ± 0.12	0.91 ± 0.51	1.03 ± 0.22	0.932
SOD, U/mL	113.09 ± 7.18	103.31 ± 9.61*	113.13 ± 3.60**	114.36 ± 5.96**	0.019
hs-CRP, pg/mL	10.30 ± 0.90	13.66 ± 2.44*	11.61 ± 2.17	9.57 ± 0.15**	0.002
MDA, nmol/mL	9.22 ± 1.84	12.06 ± 1.42*	10.94 ± 2.31	9.81 ± 1.49**	0.012
BNP, pg/mL	1842.65 ± 444.34	2264.07 ± 514.07	2153.04 ± 545.66	1975.91 ± 230.97	0.239

Values are expressed as mean ± SD

*TC* total cholesterol, *LDL-C* low-density lipoprotein cholesterol, *HDL-C* high-density lipoprotein cholesterol, *hs-CRP* high-sensitivity C-reactive protein, *MDA* malondialdehyde, *SOD* superoxide dismutase, *BNP* brain natriuretic peptide

* Compared with the control group, p < 0.05

** Compared with the DM group, p < 0.05

### Left atrial fibrosis

**Fig. 2:**
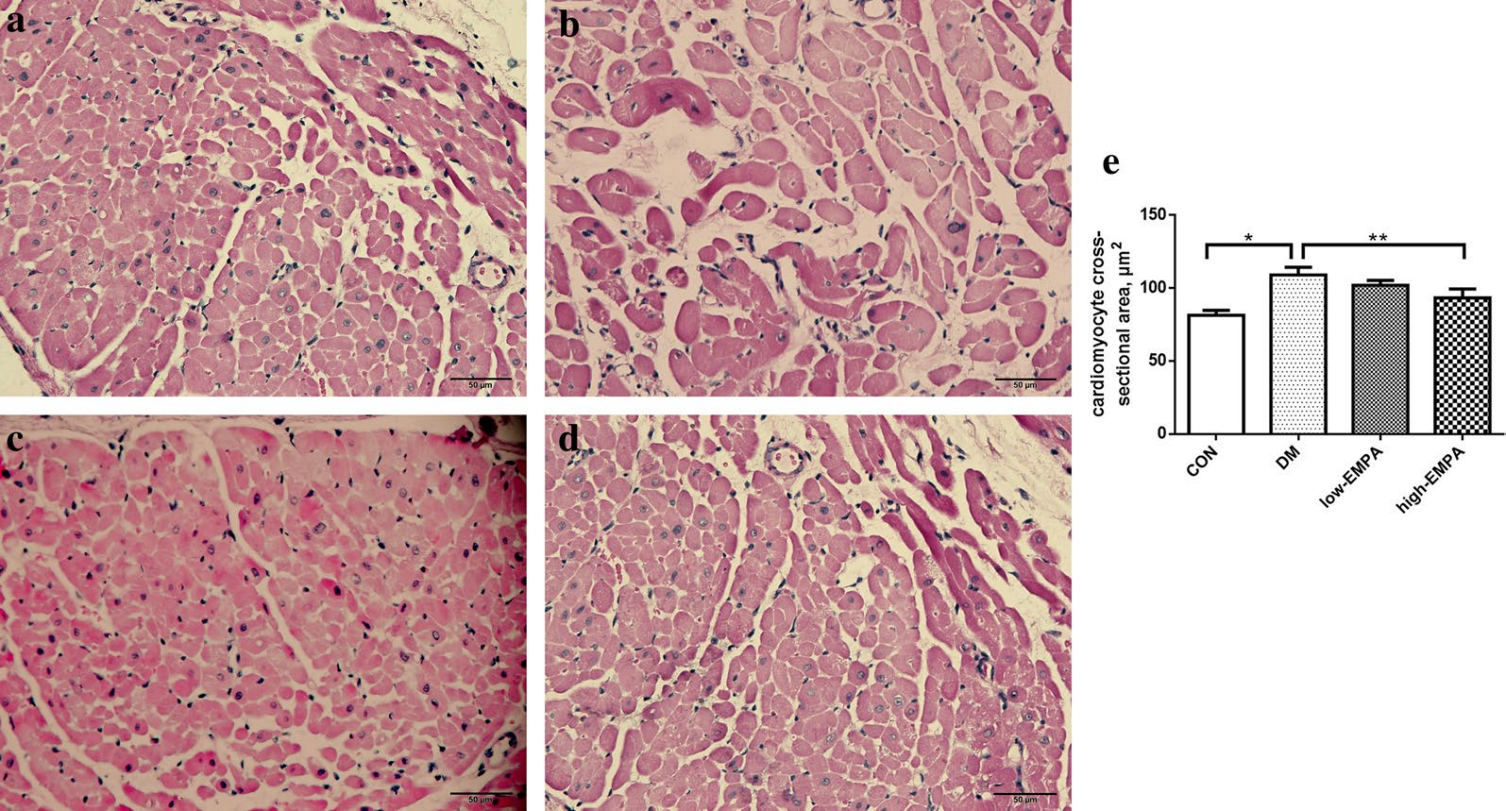
Left atrial cardiomyocyte mean cross-sectional area in the four study groups. **a** Control group; **b** diabetes mellitus (DM) group; **c** low-dose empagliflozin group; **d** high-dose empagliflozin group; **e** Illustrates cardiomyocyte cross-sectional area. Each group included 6–8 rats. *Compared with the control group, p < 0.05; **Compared with the DM group, p < 0.05

**Fig. 3:**
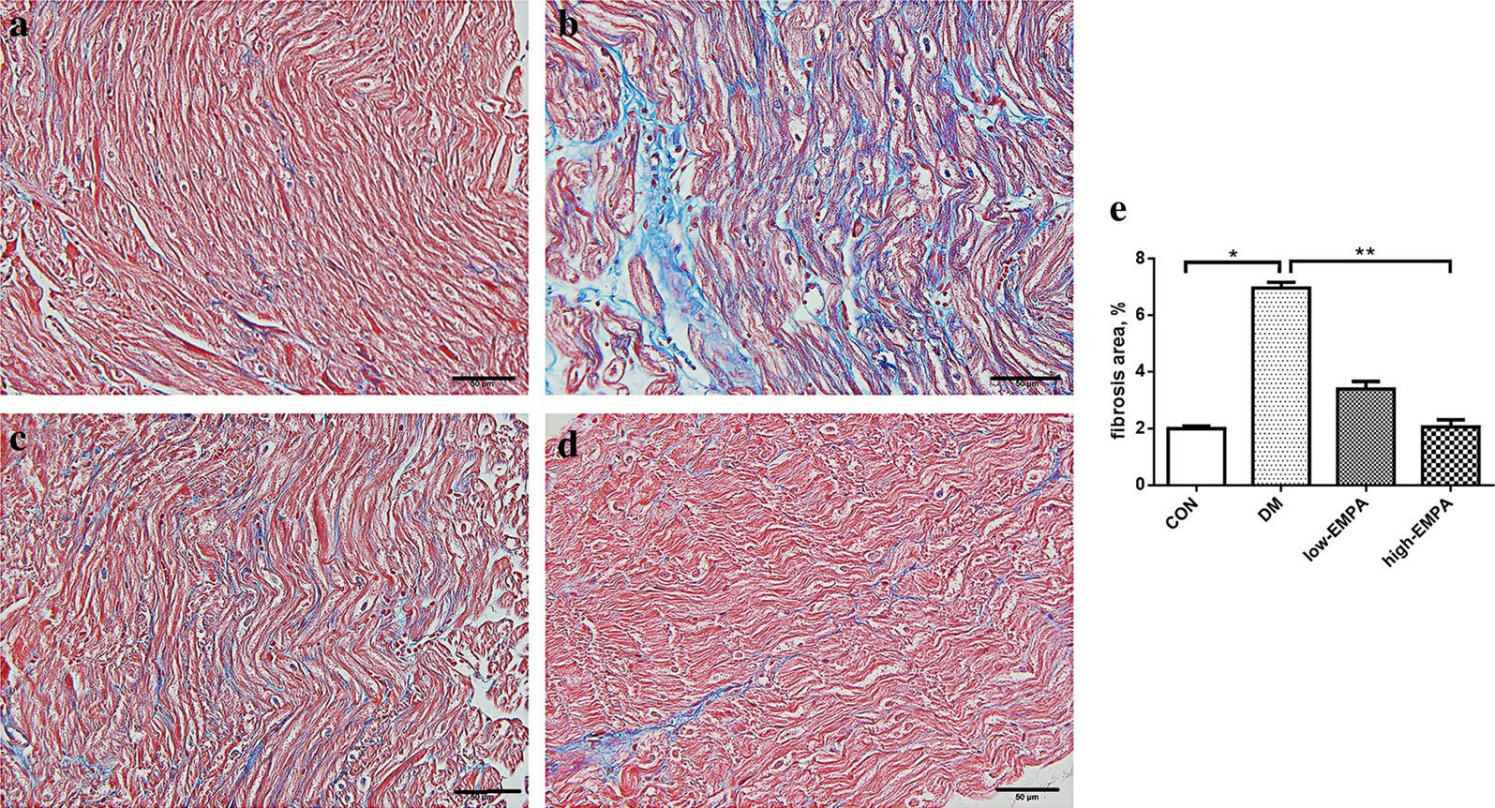
Left atrial interstitial fibrosis in the study groups. **a** Control group; **b** diabetes mellitus (DM) group; **c** low-dose empagliflozin group; **d** high-dose empagliflozin group; **e** Illustrates the quantitative ratio of the area of fibrosis to the area of the reference area. Each group included 5–8 rats.*Compared with the control group, p < 0.05; **Compared with the DM group, p < 0.05

Figures 2 and 3 showed the representative histological sections from the left atrium in the four study groups. Compared with the control group, extensive interstitial fibrosis and increased cross-sectional areas of atrial cardiomyocytes were observed in the DM group (p < 0.05), which were attenuated by the treatment of high-dose of empagliflozin (p < 0.05). The extent of attenuation was greater in the high-dose empagliflozin group compared to the low-EMPA group.

### Isolated heart electrophysiology

**Fig. 4:**
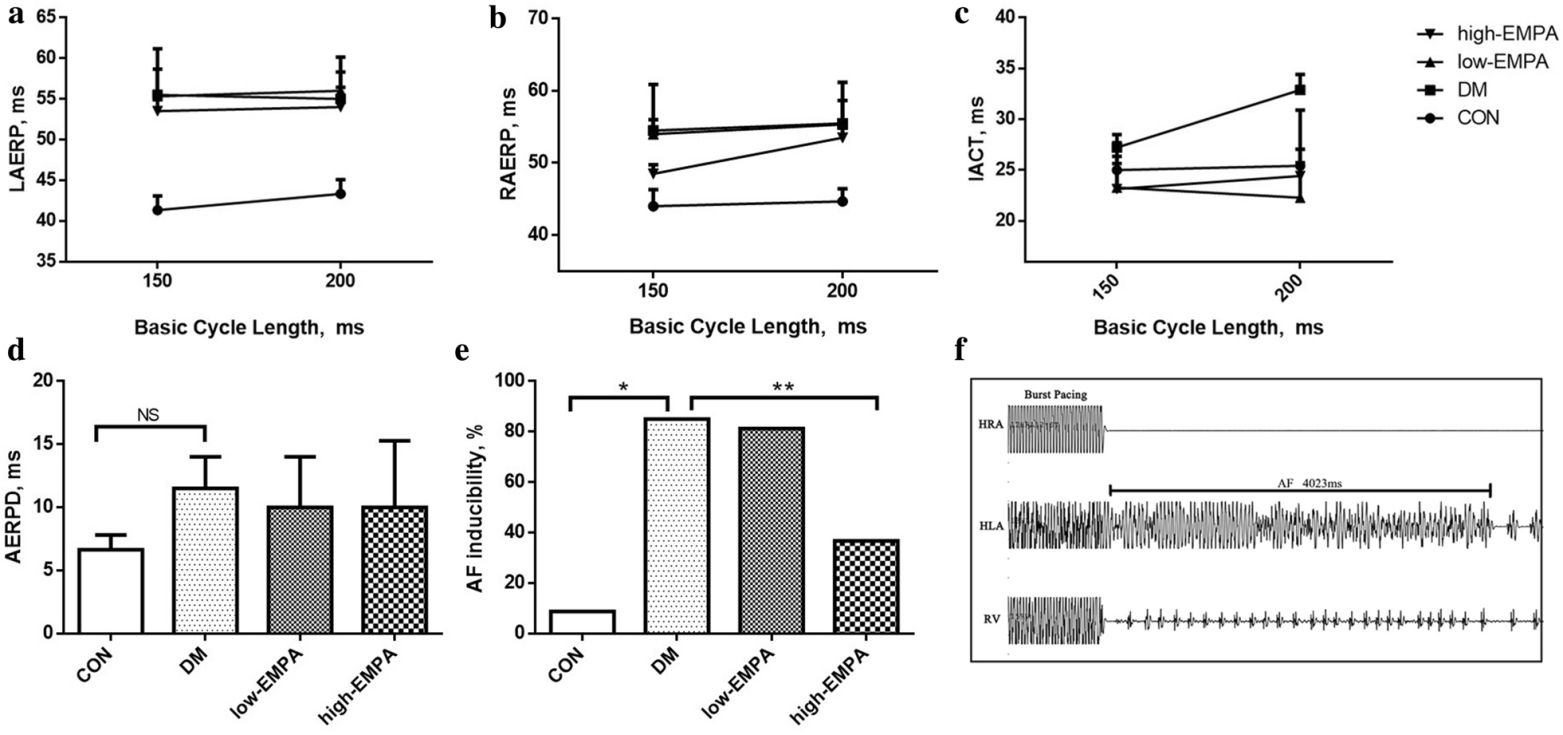
Electrophysiological measurements and atrial fibrillation (AF) induction rate. **a**–**c** Left atrium effective refractory period (LAERP), right atrium effective refractory period (RAERP), interatrial conduction time (IACT), at basic cycle lengths of 150, 200 ms. **d** Atrial effective refractory period dispersion (AERPD) of the 4 groups. **e** The inducibility of AF in the four study groups. **f** Representative AF episodes induced by burst pacing. *Compared with the control group, p < 0.05; **compared with the diabetes mellitus (DM) group, p < 0.05. Each group included 5 rats. *HLA* high left atrium, *HRA* high right atrium, *RV* right ventricle

The results of the electrophysiology studies are presented in Fig. 4. There was no difference in high left or high right atrial effective refractory periods (HLAERP and HRAERP) between the four study groups (p > 0.05). IACT in the DM group was significantly longer than those of the control group, and this prolongation was prevented by empagliflozin treatment (p < 0.05). The incidence of AF inducibility was significantly increased from 8.75 to 85%. Whilst low-dose empagliflozin treatment did not significantly reduce inducibility (81.3%), high-dose empagliflozin significantly reduced it to 36.8% (Fig. 4e).

### Mitochondrial respiratory function and membrane potential

**Fig. 5:**
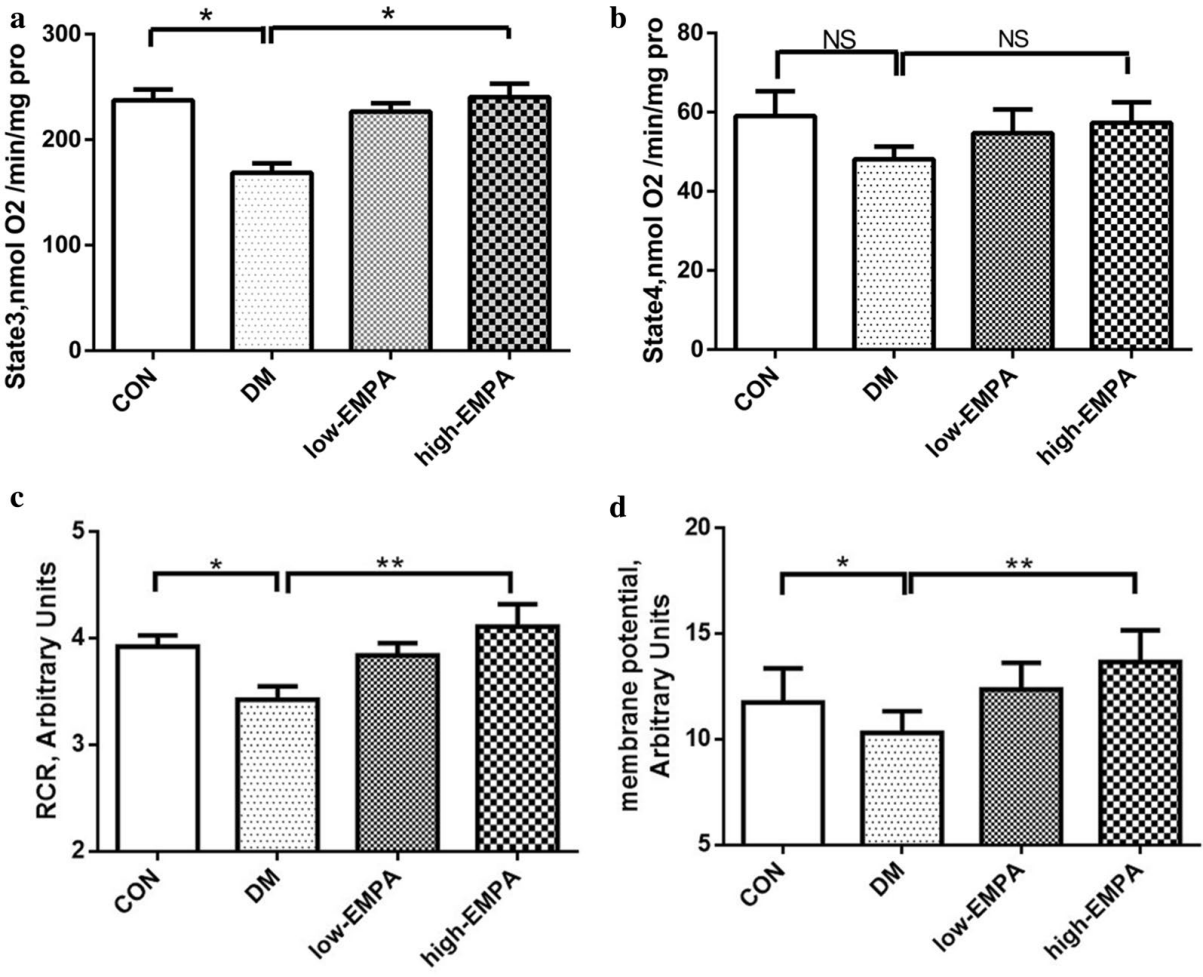
Effects of EMPA on mitochondrial state 3 respiration (**a**), state 4 respiration (**b**), respiratory control ratio (RCR) (**c**), and mitochondrial membrane potential (**d**). *Compared with the control group, p < 0.05; **compared with the diabetes mellitus (DM) group, p < 0.05. Each group included 5 rats

Compared to the control group, state 3 respiratory rate in the DM group was significantly lower (p < 0.05) whilst state 4 respiratory rate was not altered (p > 0.05), leading to a significantly reduced respiratory control ratio (p < 0.05) (Fig. 5a–c). This was accompanied by a reduction in the mitochondrial membrane potential (p < 0.05) (Fig. 5d). Whilst there was a tendency of low-dose empagliflozin treatment towards improving state 3 respiration rate and mitochondrial membrane potential, these changes were not significant (p > 0.05). By contrast, empagliflozin at the high-dose significantly restored both parameters to values that were indistinguishable from control values.

### Protein levels of mitochondrial biogenesis-related protein

**Fig. 6:**
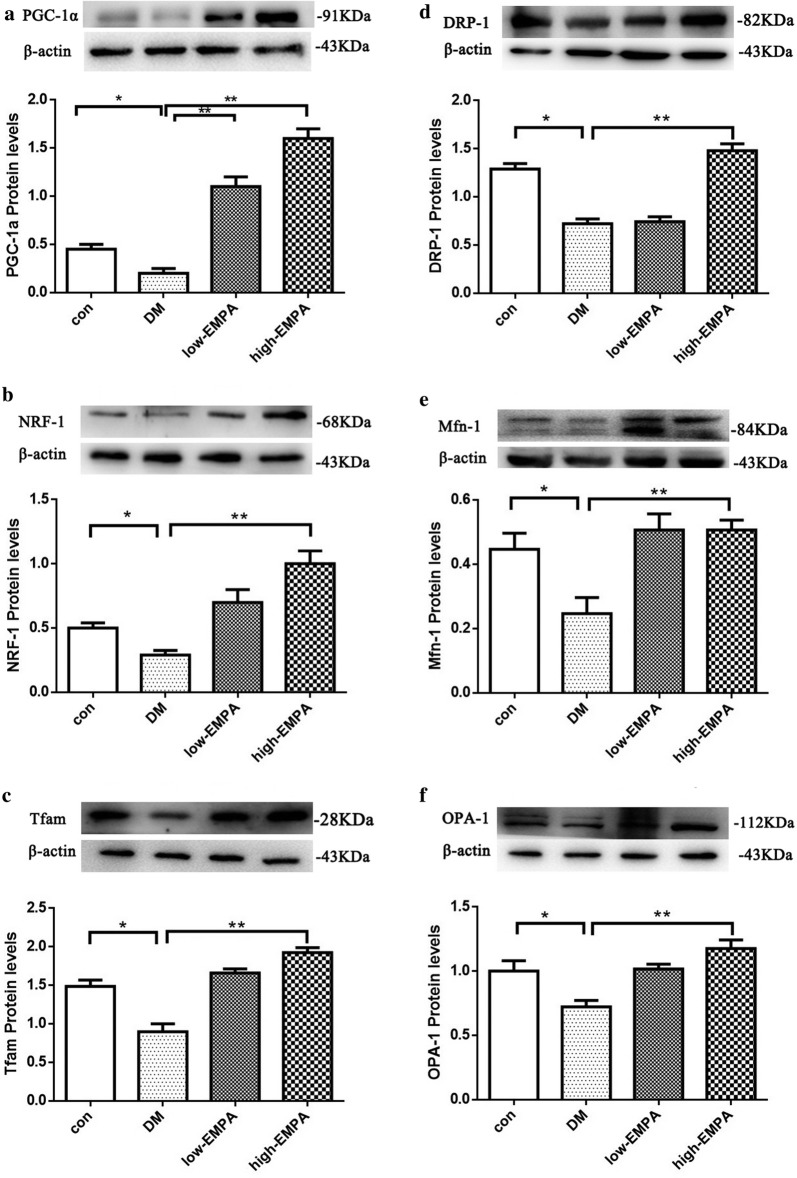
Expression levels of mitochondrial biogenesis, division and fusion protein in left atrial tissue estimated by western blot. **a**–**f** Peroxisome proliferator-activated receptor-c coactivator 1a (PGC-1a), nuclear respiratory factor-1 (NRF-1), mitochondrial transcription factor A (Tfam), dynamin-related protein 1(DRP-1), mitofusin 1(Mfn-1) and optic atrophy 1(OPA-1) protein levels in the four study groups. *Compared with the control group, p < 0.05; **Compared with the diabetes mellitus (DM) group, p < 0.05. Each group included 3–5 rats

In comparison to the control group, the protein levels of PGC-1a, NRF-1, and Tfam were significantly reduced in the DM group (Fig. 6a–c; p < 0.05). Treatment with empagliflozin increased the expression of PGC-1a, NRF-1 and Tfam, suggesting that empagliflozin can improve the mitochondrial biogenesis of atrium myocytes, which was depressed by DM. The extent of amelioration was greater in the high-dose empagliflozin group compared to the low-dose group.

### Protein levels of part of the mitochondrial division and fusion protein

The expression levels of mitochondrial division proteins dynamin-related protein 1 (DRP-1) and fusion protein mitofusin 1 (Mfn-1), in addition to optic atrophy 1 (OPA-1), were lower in the DM group compared to controls. EMPA treatment increased the protein expression of DRP-1, Mfn-1 and OPA-1 (Fig. 6d–f; p < 0.05), with a greater increase in the high-dose empagliflozin group than in low-dose group. Surprisingly, no significant effects were detected for DRP-1 in the low-dose empagliflozin group.

## Discussion

The present study is the first to identify beneficial effects of the SGLT-2 inhibitor, empagliflozin, on AF in a diabetic rat model of HFD + STZ. The major findings of this study are as follows: (1) empagliflozin attenuated DM-induced atrial structural remodeling such as LA interstitial fibrosis, atrial myocyte hypertrophy and effectively suppressed serum MDA, hs-CRP levels in diabetic rats; (2) it effectively prevented DM-induced atrial electrical remodeling (indicated by IACT) and AF inducibility; (3) it ameliorated atrial mitochondrial respiratory dysfunction, preserved mitochondrial membrane potential, improved the mitochondrial biogenesis, and may regulated mitochondrial fusion and division functions of atrial myocytes in diabetic rats.

### Atrial remodeling and diabetes mellitus

AF and DM are intertwined disorders that are linked through oxidative stress and inflammation. DM exacerbates atrial electrical and structural remodeling, thereby facilitating AF initiation and maintenance [13, 14]. Conversely, hence the presence of AF indicates an increased risk in cardiovascular event and mortality amongst DM patients [4, 15, 16]. The initial stage of DM-induced myocardial changes is characterized by increased fibrosis and stiffness, which is reflected by reduced early diastolic filling, increased atrial filling and enlargement, and elevated LV end-diastolic pressure [17, 18]. We further demonstrated that LAD, IVST, LVPWT and LVEDP were significantly increased in the DM rats compared with controls, which was consistent with results of the previous studies.

Although the precise pathophysiological mechanisms of DM-induced AF have not been fully elucidated, structural and electrical remodeling are two major contributors to the AF substrate, which refers to the factors that predispose to arrhythmogenesis [19]. Atrial interstitial fibrosis and replacement fibrosis are hallmarks of arrhythmogenic structural remodeling, producing electrical conduction heterogeneity and disturbance and eventually AF [16, 20].

Previously, our team has employed different animal models for evaluating the pathophysiological mechanisms that underlie adverse cardiac remodeling in diabetes. For example, alloxan-induced diabetic rabbits exhibit atrial interstitial fibrosis and increased AF inducibility, which are associated with prolonged AERP dispersion and IACT [14, 15]. In this study, we used the HFD + STZ injection to induce diabetes. Some animals did not meet the criteria of developing diabetes after a single injection of STZ. In these animals, a second dose was injected. Whilst this may increase phenotypic variability between the experimental animals, this double injection approach has been used by other research groups. A previous study used high-fat diet combined with 30 mg/kg STZ twice injection presented a typical characteristic of T2DM as insulin resistance, hyperglycemia, and represented a stable disease model of T2DM [16].

Data from the present study further support the notion that AF is associated with atrial interstitial fibrosis and electrical conduction heterogeneity. Moreover, indirect evidence supporting the involvement of increased inflammation and oxidative stress in DM-induced atrial remodeling is presented by our data on the serum biochemical examination, where the content of lipid oxidation products, MDA and hs-CRP, are sharply raised while SOD is reduced in the circulation of diabetic rats. Thus, evidence from this study supports the notion that DM contributes to the induction of AF through the promotion of atrial remodeling.

### Mitochondrial dysfunction, atrial remodeling and diabetes mellitus

Recently, several studies in DM models have reported mitochondrial function dysfunction in the myocardium as an important pathological change [11, 18, 19]. Cardiomyocytes contain a relatively large number of mitochondria and mitochondrial oxidative phosphorylation provides 90% of intracellular ATP production in cardiomyocytes. An emerging body of evidence shows that mitochondria and nicotinamide adenine dinucleotide phosphate (NADPH) oxidase are the predominant mechanisms by which ROS is generated in the diabetic heart [21]. Hyperpolarization of the mitochondrial membrane and impaired mitochondrial function promote mitochondrial ROS production. ROS can damage mitochondrial membrane structure and oxidize lipids to yield reactive lipid peroxidation products [22]. Moreover, ROS can activate mitochondrial uncoupling, leading to reduce cardiac efficiency. Altered mitochondrial Ca^2+^ handling further promotes mitochondrial respiratory dysfunction, which ultimately leads to cell death under increased oxidative stress. Our previous work has found that increased mitochondrial reactive oxygen species production rate, depolarized mitochondrial membrane potential, and mitochondrial swelling in diabetic rabbits [23]. In the present study, we extend these findings by reporting abnormal mitochondrial membrane potential and mitochondrial respiratory control ratio, increased ROS production, as well as abnormal expression levels mitochondrial proteins in the DM rats.

Mitochondrial abnormalities may play an important role for the increased propensity for AF in DM. Previous studies have demonstrated that abnormal mitochondrial structure and function prompted atrial structural and electrical remodeling [24, 25]. Damage to mitochondrial function leads to the increasing extracellular matrix, causing further contractile dysfunction, hypertrophy and cardiac fibrosis [26]. In addition, mitochondrial dysfunction was associated with altered cardiac electrical properties, giving rise to action potential heterogeneities in conduction or repolarization in both the ventricles and the atria [23, 27]. In this present study, we observed higher AF inducibility in the DM group, and markedly reduced by EMPA. These findings complement previous studies focusing on alterations in ventricular structure and electrical function in diabetic animals and the effects of anti-diabetic treatment upon these. For example, empagliflozin was shown to improve left ventricular diastolic function a genetic mouse model of T2DM [20]. Furthermore, the SGLT-2 inhibitor dapagliflozin was shown to suppress prolonged ventricular-repolarization through augmentation of mitochondrial function and protection against the generation of reactive oxygen and nitrogen species in insulin-resistant metabolic syndrome rats [28].

PGC-1a is a crucial promoter of mitochondrial biogenesis, regulated by adenosine monophosphate kinase (AMPK) and induced by NRF-1 and Tfam [29, 30]. There is evidence that the SGLT-2 inhibitor attenuates the up-regulation of the cardiac Na^+^/H^+^ exchanger (NHE) in vitro in mouse cardiac fibroblasts stimulated with lipopolysaccharides (LPS) via AMPK activation [31]. In our study, we found that the protein level of PGC-1a, NRF-1, and Tfam expressed in LA tissue were reduced in the DM group, which corresponds to the impaired mitochondrial biogenesis in DM described by recent studies. Moreover, the protein expression level of DRP-1 and Mfn-1, mitochondrial division and fusion proteins respectively, in addition to OPA-1, are also lower in DM group, suggesting that both the quality and function of atrial myocardial mitochondria is reduced in DM, resulting in suppressed mitochondrial fusion and division.

### SGLT-2 inhibitor, empagliflozin and potential cardiac beneficial effects

SGLT-2 is a sodium–glucose cotransporter exclusively expressed in the kidney. They are mostly localized in the brush border membrane of the proximal tubule epithelial cells in the S1 segment of the proximal convoluted tubule [32, 33]. SGLT-2 expression is significantly increased in diabetic humans, rats, and db/db mice [34–36]. It is correlated with glomerular hyperfiltration, increased glucose reabsorption, and elevated plasma glucose [37]. Conversely, SGLT-2 inhibition leads to natriuresis, osmotic diuresis, plasma volume contraction. Recognizing the physiological effects of SGLT-2, SGLT-2 inhibitors have emerged as a new class of plasma glucose-lowering medication. Besides from beneficial effects on parameters such as glucose concentration, weight, blood pressure and albuminuria, SGLT-2 inhibitors potentially reduce the risk of cardiovascular mortality and HF [38]. Recently, several large-scale randomized controlled trials, the EMPA-REG OUTCOME, DECLARE-TIMI and CANVAS, provided evidence that SGLT-2 inhibitors reduce the risk of cardiovascular events significantly when compared to the use of placebo [4, 5, 39]. Other teams have contributed to the understanding of cardiac remodeling in diabetic rat models using SGLT-2 inhibitors [40–45]. The cardiac protection effect of SGLT-2 inhibitor was not only in DM, still in cardiorenal syndrome (CRS) animals. Yang et al. [46] found that compared with CRS animals, LVEF was remarkably preserved and LV remodeling was substantially suppressed in CRS animals treated by EMPA. The finding may support the results of a previous clinical trial [4]. In addition, EMPA also ameliorates type 2 diabetes-induced ultrastructural remodeling of the neurovascular unit and neuroglia in the female db/db mouse [47].

Several hypotheses about the potential mechanisms of SGLT‑2 inhibitors responsible for cardioprotection have been identified [48, 49]. These include prevention of (i) cardiac inflammation and oxidative stress, (ii) apoptosis, (iii) ionic homeostasis, and mitochondrial dysfunction. Indeed, Durak et al. [28] suggested a new insight into another SGLT-2 inhibitor DAPA-associated cardioprotection, including suppression of prolonged ventricular-repolarization through augmentation of mitochondrial function and oxidative stress. Furthermore, Lee et al. [50] have found that EMPA could change Ca^2+^ regulation, late Na^+^ and Na^+^/H^+^-exchanger current, and electrophysiological characteristics in DM cardiomyopathy.

To the best of our knowledge, this study is the first to examine the protective effects SGLT-2 inhibitor on the diabetic atria and explore the roles of mitochondrial dysfunction as an underlying pathogenic mechanism driving this atrial remodelling. Our study demonstrated that the SGLT-2 inhibitor, EMPA, have favorable effects in ameliorating arrhythmic substrate, improving electrophysiological abnormalities, and reducing AF inducibility. Meanwhile, we also found that SGLT-2 inhibitors increased LA tissue mitochondrial respiration function and the protein expression levels of PGC-1a, NRF-1, Tfam, DRP-1, Mfn-1 and OPA-1, indicating that EMPA can improve the impaired mitochondrial function in DM. With reduced mitochondrial impairment and atrial remodeling in DM, the present study provides evidence for potential clinical use of EMPA upon the prevention of DM-induced AF.

However, it is not clear the relative contributions of direct cardiac versus systemic effects towards cardioprotection mediated by SGLT-2 inhibition. For example, DM is associated with heart failure, and SGLT-2 inhibitors could reduce the risk of cardiovascular mortality by preventing HF [38, 39]. Indeed, there are close and complex links between heart failure with preserved ejection fraction (HFpEF) and AF, with shared risk factors and the higher AF incidence in HFpEF may independently contribute to poor clinical outcomes [51]. In this study, we discovered that EMPA improved diastolic dysfunction on diabetic rats. It is likely that the improvement in HF partly contributed to reduced risk of AF inducibility by high-dose EMPA. In our animal model, both glucose and lipid profiles were reversed by EMPA, and thus the cardiac protective effects must have been partially mediated by alterations in lipid and glucose levels. To dissect the relative contributions of glucose-dependent and glucose-independent effects, further studies need to examine the effects of empagliflozin on cardiac function in non-diabetic models such as heart failure or hypertension models. Moreover, we found direct amelioration of atrial electrical and structural substrate and prevention of mitochondrial dysfunction by empagliflozin, and thus direct cardiac effects may operate in this setting.

In addition, Na^+^ and Ca^2+^ handling abnormalities contribute to cardiac contractile dysfunction, as well to the perpetuation and progression of AF. Baartscheer et al. [52] demonstrated the direct myocardial effects of empagliflozin on Ca^2+^ and Na^+^ concentrations independently of SGLT-2 activity. Thus, in isolated ventricular myocytes from rabbits and rats incubated with empagliflozin for 3 h, empagliflozin decreased cytoplasmic Na^+^ ([Na^+^]_c_) and Ca^2+^ ([Ca^2+^]_c_) and also increased mitochondrial Ca^2+^ concentration ([Ca^2+^]_m_). Empagliflozin can exert effects by decreasing myocardial [Na^+^]_c_ and [Ca^2+^]_c_ and increasing [Ca^2+^]_m_ through the inhibition of the Na^+^/H^+^ exchanger (NHE) directly [46]. In addition, it has been shown that empagliflozin improved LV diastolic function by increasing sarcoplasmic endoplasmic reticulum Ca^2+^-ATPase (SERCA2a) activity in diabetic mice [53]. Hence, further in-depth study and important knowledge gaps need to be addressed by a multidisciplinary experimental and computational approach to investigate the specific roles of NHE, Na^+^ and Ca^2+^ ionic concentrations in atrial muscle, as has been performed for skeletal muscle [54].

## Limitations

Several limitations should be considered for the present study. Firstly, the SGLT-2 levels in plasma and atrial cardiomyocytes were not quantified. Secondly, the dosage of empagliflozin used was greater than the clinical dose used in humans to ensure sufficient SGLT-2 inhibition. Thirdly, empagliflozin did not reduce blood pressure, and considered that the time of drug intervention was short in our study. Studies have found that systolic blood pressure was no change or dropped 1–2 mmHg at 8 weeks, while 3–4 mmHg at 12 weeks [55, 56]. Fourthly, serum angiotensin-II level, which is associated with high blood pressure, was not measured. Empagliflozin ameliorated cardiac hypertrophy in prediabetic metabolic syndrome rat, independently of blood pressure lowering effect, in association with the attenuation of cardiac oxidative stress and inflammation [55]. Fifthly, SGLT-2 inhibitors not only lower blood glucose levels, but also have diuretic effects, but the amount of diuresis was not examined in our study. Sixthly, SGLT-2 inhibition with Empagliflozin has been shown attenuate mitochondrial oxidative mitochondrial DNA damage and also restore mitochondrial numbers in rats with heart failure [57]. It is possible that these mechanisms could also underlie the improvements in mitochondrial function observed in our study. Detailed analysis of these mechanisms are, however, beyond the scope of this investigation. In addition, ionic currents will be measured in the presence and absence of empagliflozin in atrial myocytes isolated from normal, diabetic, hypertensive and failing hearts, to test the hypothesis that empagliflozin can modulate ion channel activity via glucose-dependent and glucose-independent mechanisms. Seventhly, the same dose of STZ was injected again in rats with blood glucose level that did not meet the diagnostic criteria, which might increase the variation of animals in the same group. However, this double injection approach has been used by other research groups. A previous study used high-fat diet combined with 30 mg/kg STZ twice injection presented a typical characteristic of T2DM as insulin resistance, hyperglycemia, and was a stable animal model of T2DM [17]. Finally, due to species differences between rats and humans, our work needs to be interpreted with caution. A recent meta-analysis pooling data from sixteen studies reported the incidence of AF comparing between patients under the use of SGLT-2 inhibitors and placebo (SGLT-2 inhibitors, 7191 patients, 17 events; placebo, 3321 patients, 13 events) [58]. The overall SGLT-2 inhibitor group, as well as the canagliflozin, empagliflozin and dapagliflozin subgroups had no significant impact on the incidence of atrial fibrillation. The fundamental pathogenesis of AF may differ between species due to differences in heart rate, atrial size, and atrial ion channel expression.

## Conclusions

Empagliflozin can prevent atrial structural and electrical remodeling, improve mitochondrial function and mitochondrial biogenesis in T2DM, hence may be potentially used in the prevention of DM-related AF.

## Data Availability

The datasets used and/or analyzed during the current study are available from the corresponding author on reasonable request.

## Abbreviations

SGLT-2: sodium–glucose co-transporter-2
DM: diabetes mellitus
T2DM: type II diabetes mellitus
AF: atrial fibrillation
EMPA: empagliflozin
HFD: high-fat diet
STZ: streptozocin
ISO: isoflurane
EDV: end-diastolic volume
ESV: end-systolic volume
LVEDD: left ventricular end-diastolic dimension
LVESD: left ventricular end systolic dimension
PGC-1α: peroxisome proliferator-activated receptor-c coactivator 1α
NRF-1: nuclear respiratory factor-1
Tfam: mitochondrial transcription factor A
EMPA-REG OUTCOME: EMPAgliflozin Removal of Excess of Glucose OUTCOME
CVD: cardiovascular disease
HF: heart failure
CANVAS: CANagliflozin cardiovascular Assessment Study
SD: Sprague–Dawley
ig: intragastric administration
CON: control
LA: left atrial
LVPWT: left ventricular posterior wall thickness
IVST: interventricular septal thickness
LVEDD: left ventricular end-diastolic dimension
LVESD: left ventricular end systolic dimension
LVEF: left ventricular ejection fraction
ECG: electrocardiograph
SBP: aortic systolic blood pressure
DBP: diastolic blood pressure
MBP: mean blood pressure
TC: total cholesterol
TG: triglycerides
LDL-C: low-density lipoprotein cholesterol
HDL-C: high-density lipoprotein cholesterol
Cr: creatinine
BNP: brain natriuretic peptide
hs-CRP: high-sensitivity C-reactive protein
MDA: malondialdehyde
SOD: superoxide dismutase
HE: hematoxylin and eosin
HR: heart rate
IACT: interatrial conduction time
HRAERP: high right atrial effective refractory period
HLAERP: high left atrial effective refractory period
ADP: adenosine diphosphate
ATP: adenosine triphosphate
Dw: mitochondrial membrane potential
PVDF: polyvinylidene fluoride
Mfn-1: fusion protein mitofusin 1
OPA-1: optic atrophy 1
DRP-1: dynamin-related protein 1
FFA: free fatty acid
ROS: reactive oxygen species
AMPK: adenosine monophosphate kinase
NHE: Na^+^/H^+^ exchanger
AP: action potential
LPS: lipopolysaccharides
